# Child Life under Queen Victoria

**Published:** 1897-02-13

**Authors:** 


### CHILD LIFE UNDER QUEEN VICTORIA.

### I.—PRELIMINARY: THE DOMESTIC SYSTEM.

In looking back over the sixty years during which Queen Victoria has sat upon the throne of Britain, one naturally tries to sum up in a phrase the main characteristics of the longest reign our history has known. But to do so is impossible. So full of change has the period been that one might with equal truth define it as the most distinguished England has known in several developments. We propose to deal with only one feature of Her Majesty's reign, though in doing so we must frequently speak of another. We cannot avoid speaking of the great expansion of trade and development of manufacture consequent on the employment of steam to drive machinery, when we treat of child-labour, and the prominent place which children have occupied in the statute book during the reign of Victoria. It was the great development of the Factory System under the influence of steam power that created that unprecedented demand for children's labour which compelled legislation, and the two can hardly be considered apart.

Of the factory system—the modern factory system—we must speak, and, to a great extent, we must speak evil. It is only fair, therefore, to show, as a preliminary, that it did not, as some have pretended, come like a monster to sweep away an idyllic system to which we ought to return. It was no new thing sixty years ago for children to be at work at an age when, according to our modern notions, they ought to have been at school. In some subsidiary part of spinning, weaving, straw plaiting, lace making—every employment that was carried on at home—children had always been employed. Nay, the factory itself was not a new thing, nor child-labour in it. "Jack of Newbury,"* a famous cloth-weaver of Tudor times, employed more than a thousand persons in his works, among whom were two hundred "pretty boys," engaged in "making quills with mickle joy" (we doubt the joy) and a hundred and fifty "children of poor silly men" who were employed in picking wool. We omit the six scullion boys who helped in the kitchen—for Jack fed his workpeople, and seems to have fed them well—and the "poor children that did stay to turn the broaches every day," as not belonging to the factory proper. That still leaves a third of Jack of Newbury's workers who were children. The difference between this and a modern factory is primarily, of course, that it was all handicraft instead of power-loom work, but secondly—and this is the moral differentiation—in it the labour of children was subsidiary to that of adults, whereas in the modern factory in its early days, before the hand of the Legislature interfered, the children to a great degree superseded their seniors. The economic consequences of this to the workers were serious; they were in a manner forced to let their own children undersell them in the labour market, for if they rebelled pauper apprentices were imported; but the moral consequences could not but be equally serious. The parent became the task-master or the task-master's coadjutor, and nothing can be more demoralising to a man, more destructive of all natural feeling and sense of duty, than such a reversal of natural relations as is implied in the child supporting the parent.

But child-labour was not, as we have said, a new thing, and it is doubtful if in the earlier days the child-worker's lot was happier or his conditions of work more wholesome than in the factories. The poets gave us idyllic pictures of family life and work, but prosaic observers cast a different light on the question. Dr. Cooke Taylor, in his history of the silk, cotton, and woollen manufactures, declares, indeed, that infant labour "was at its worst and greatest height before anyone thought of a factory." Mr. Carroll D. Wright, the Special Commissioner sent from the United States to investigate the origin and working of the factory system in Britain, declares that "the hut of the domestic worker was occupied day and night by a class which has not found, and cannot find, its like under the factory system." Again: "The weaver's family lived and worked without comfort, conveniences, good food, good air, and without much intelligence."

So much for the conditions; we have convincing testimony as to the age when work began. We are told of Samuel Crompton, the inventor of the spinning mule, that "his little legs became accustomed to the loom almost as soon as they were long enough to touch the treadles."* But his own experience did not induce him to make his children's lives easier. George Crompton, his eldest son, thus testifies: "I recollect that soon after I was able to walk I was employed in the cotton manufacture." (Think of an infant of two or three years old contributing to the family maintenance!) My mother used to bat the cotton wool on a wire riddle. It was then put into a deep brown mug with a strong ley of soapsuds. My mother then tucked up my petticoats about my waist, and put me into the tub to tread upon the cotton at the bottom. When a second riddleful was batted I was lifted out; it was placed in the mug, and I again trod it down. This process was continued until the mug became so full that I could no longer safely stand on it, when a chair was placed beside it, and I held on by the back." When we learn that parents put mere babies to such work as this we do not need to believe that manufacturers were fiends in human form, because they made their child workers labour hard and long. The conception of the rights of children had not yet entered human consciousness. Even benevolent observers were misled by the apparent economic benefit, and did not think of the effects on the health and welfare of the children. Defoe tells how in the eastern counties "the very children after four or five years of age could every one earn their own bread," and Richard Braithwaite, of Kendal, praises the "worthy care" of the inhabitants of that town "to see their very young children put to worke," evidently regarding these as model parents. This "worthy care" continued till the rise of the factory system. Indeed, it more or less continues still, though other legislation, not directly industrial, has to some extent interfered with the subjection of young children to it. But we have found out its nature. We know now that the conditions of work in even bad factories are better than in the average home. We know that while the work hours in the factory are long, work hours in the home may be endless. We no longer style such work by the pleasing name of "home industry"; we call it "the sweating system."

* John Smallwood, also called John Wynchcombe or Whitcombe, from the name of the village in which he was born, and Jack of Newbury from the town in which his factory was situated. For an interesting metrical account of his early weaving shop see "The Modern Factory System," by R. Whately Cooke Taylor, F.R.Hist.S., H.M. Inspector of Factories. (London: Kegan Paul, Trench, Trubner, and Co.) Jack of Newbury is included by Fuller in his English "Worthies."

* For this and following quotation see French's "Life of Crompton."

